# Lifestyle and socio-economic inequalities in diabetes prevalence in South Africa: A decomposition analysis

**DOI:** 10.1371/journal.pone.0211208

**Published:** 2019-01-30

**Authors:** Chipo Mutyambizi, Frederik Booysen, Andrew Stokes, Milena Pavlova, Wim Groot

**Affiliations:** 1 Population Health, Health Systems and Innovation, Human Sciences Research Council, Pretoria, South Africa; 2 University of the Witwatersrand, Johannesburg, South Africa; 3 Department of Global Health, Boston University School of Public Health, Boston, Massachusetts, United States of America; 4 Department of Health Services Research; CAPHRI, Maastricht University Medical Centre, Faculty of Health, Medicine and Life Sciences, Maastricht University, Maastricht, The Netherlands; Sciensano, BELGIUM

## Abstract

**Background:**

Inequalities in diabetes are widespread and are exacerbated by differences in lifestyle. Many studies that have estimated inequalities in diabetes make use of self-reported diabetes which is often biased by differences in access to health care and diabetes awareness. This study adds to this literature by making use of a more objective standardised measure of diabetes in South Africa. The study estimates socio-economic inequalities in undiagnosed diabetes, diagnosed diabetes (self-reported), as well as total diabetes (undiagnosed diabetics + diagnosed diabetics). The study also examines the contribution of lifestyle factors to diabetes inequalities in South Africa.

**Methods:**

This cross sectional study uses data from the 2012 South African National Health and Nutrition Examination Survey (SANHANES-1) and applies the Erreygers Concentration Indices to assess socio-economic inequalities in diabetes. Contributions of lifestyle factors to inequalities in diabetes are assessed using a decomposition method.

**Results:**

Self-reported diabetes and total diabetes (undiagnosed diabetics + diagnosed diabetics) were significantly concentrated amongst the rich (CI = 0.0746; *p* < 0.05 and CI = 0.0859; *p* < 0.05). The concentration index for undiagnosed diabetes was insignificant but pro-poor. The decomposition showed that lifestyle factors contributed 22% and 35% to socioeconomic inequalities in self-reported and total diabetes, respectively.

**Conclusion:**

Diabetes in South Africa is more concentrated amongst higher socio-economic groups when measured using self-reported diabetes or clinical data. Our findings also show that the extent of inequality is worse in the total diabetes outcome (undiagnosed diabetics + diagnosed diabetics) when compared to the self-reported diabetes outcome. Although in comparison to other determinants, the contribution of lifestyle factors was modest, these contributions are important in the development of policies that address socio-economic inequalities in the prevalence of diabetes.

## Background

Non-communicable diseases (NCDs) are currently the leading cause of death globally. According to the World Health Organisation, NCDs are projected to overtake all other causes of death in Africa by the year 2030 [1]. In the last two decades, the prevalence of diabetes has increased from 4.7% in 1980 to 8.5% of the total world population in 2014 and is expected to further increase especially in lower and middle income countries [2]. Between 1990 and 2013, the years of life lost to diabetes globally have increased by 67% [3]. Historically diabetes was a burden of developed countries but a huge increase has now been reported in developing countries [2], countries that often do not have the resources for the prevention, diagnosis, treatment and management of the disease [4]. In South Africa, the International Diabetes Federation (IDF) estimates that in 2015, almost 2.3 million people had diabetes [5]. The magnitude of the diabetes burden is further reflected in the mortality and causes of death statistics, which show that diabetes has moved from being the fifth leading underlying cause of death in 2013 to being the third and second leading underlying cause of death in 2014 and 2015, respectively [6].

Research has been consistent in showing that there is an association between socioeconomic inequalities and diabetes prevalence [7–10]. Whilst earlier studies in high income countries have shown that diabetes prevalence is associated with high socio-economic status (SES), more recent findings show that it is associated with lower SES [7, 8]. On the other hand, recent findings from middle and low income countries show an association of diabetes prevalence with higher SES [9–11]. In Africa, there is a paucity of studies estimating the socioeconomic inequalities in diabetes [8], in particular studies that use the concentration index (CI) as a measure of inequality. The CI is a measure which assesses relative inequality in health. The index shows the distribution of ill-health across the income distribution or some other living standards measure [12]. Earlier studies on inequalities in illness in South Africa that have used the concentration index demonstrate that socio-economic inequalities in health exist, however the prevalence across socioeconomic status varies by disease type [13–15]. Studies that have reported on the socio-economic inequalities in diabetes show that the distribution of illness is higher among more affluent socio-economic groups [13, 15]. Ataguba et al. find that although the CI for diabetes showed that the socio-economic distribution of diabetes was greater among people in higher socio-economic groups the index has declined from 0.10 in 2002 to 0.01 in 2008 [13]. In a more recent study, Mukong et al. finds that the CI for diabetes was 0.024 in 2008 and 0.034 in 2014–2015 [15]. Both studies however relied on self-reported measures of diabetes [13, 15].

The use of self-reported data on illness without any other standardised measures is reported to result in the exclusion of undiagnosed individuals, especially those in groups with low socio-economic status [16] who might have relatively less access to diagnostic services when compared to high income groups [17]. It is estimated that over two thirds of people with diabetes in the African region are undiagnosed [5]. In South Africa, it is estimated that approximately 1.396 million people with diabetes are not diagnosed [5]. The magnitude of the unmet need for diabetes care in South Africa has also been previously analysed and reported by Stokes et al. [18]. Using the 2012 South African National Health and Nutrition Examination Survey, the authors find that close to half of individuals with diabetes were undiagnosed [18]. Poorer and less educated people tend to have relatively worse access to medical care for diabetes diagnosis when compared to the more educated and wealthier individuals [16]. As a result, the exclusion of undiagnosed diabetics may produce biased diabetes prevalence and inequality estimates.

Whilst the causes of type 1 diabetes are unknown, the risk of type 2 diabetes is determined by an interplay of factors such as ethnicity, age, socio-economic status and various lifestyle factors [2]. Lifestyle factors such as unhealthy diets, smoking, alcohol consumption and physical inactivity are particularly important for the prevention of type 2 diabetes [2, 19, 20], which is more common globally [4, 5]. The role of modifiable risk factors in explaining the inequality in diabetes has been previously investigated [15, 21, 22]. Health behaviours such as smoking and alcohol consumption explain between 33–45% of inequalities in the incidence of type 2 diabetes in the United Kingdom [21] and a third of socioeconomic inequalities in type 2 diabetes in a Swedish based study [22]. Using data from the South Africa National Income Dynamics Survey, Mukong et al. finds that smoking and alcohol consumption account for -2.4% and 2.2% of self-reported diabetes inequality in 2014–2015 [15]. The importance of addressing these risk factors is highlighted in these studies and is also entrenched in the World Health Organisation (WHO) Global Status report on non-communicable diseases [23].

Estimating inequalities in diabetes and determining the contributions of avoidable diabetes risk factors to these inequalities can help South Africa in working towards meeting the 2030 sustainable development goal 3 (SDG 3), which targets a reduction in premature deaths due to NCDs (including diabetes). This study therefore aims to (1) describe the prevalence, treatment and control of diabetes among South Africans across various socio-economic groups; (2) to determine socio-economic inequalities in the prevalence of diabetes using the CI; and (3) to examine the contribution of dietary, lifestyle and metabolic risk factors to socio-economic inequalities in diabetes prevalence by conducting a decomposition analysis. Our study makes important contributions to the body of literature on inequalities in diabetes. To the best of our knowledge this is the first South African study to make use of clinical outcomes in addition to self-reported data in the estimation of socio-economic inequalities in diabetes and the only study that allows a more in-depth analysis into the contribution of a number of lifestyle factors to inequalities in diabetes.

## Methods

### Data

Data are taken from the 2012 South African National Health and Nutrition Examination Survey (SANHANES-1)[24]. SANHANES-1 is a nationally representative survey undertaken in April to November 2012 to assess the health and nutrition status of the South African population. It is the first comprehensive national survey on NCDs in South Africa. The survey received clearance from the Research Ethics Committee (REC) of the Human Sciences Research Council (REC 6/16/11/11). Informed consent was obtained from all study participants. A stratified, multi-stage cluster sample design was employed in sampling the households to be included in the survey. The 2001 population census was used to select a total of 1 000 enumeration areas (EAs) from a database of 86 000 EAs. The selection of EAs was stratified by province and locality. In formal urban areas, the selection of EAs was further stratified by race. Based on the master sample of 1 000 EAs, a total of 500 EAs were selected based on the socio-demographic profile of South Africa. A random sample of 20 dwellings was then randomly selected from the EAs, yielding a sample of 10 000 dwellings or so-called visiting points. Out of the 8 168 valid, occupied households (the balance of 1 832 dwellings were vacant or could not be located), 6 306 households residing at these houses agreed to be interviewed (response rate = 77.2%). The dataset includes 26 806 individuals.

The SANHANES-1 survey comprised a questionnaire component and a clinical examination. Three questionnaires were administered during the survey: a household questionnaire, an adult questionnaire and a child questionnaire. In this study, the household questionnaire is the source of data for the wealth index. Information on self-reported diabetes and lifestyle factors is drawn from the adult questionnaire. The adult and child questionnaires were administered in the individual’s households. The SANHANES-1 survey did not draw a distinction between type 1 and type 2 diabetes.

Blood samples were collected during the clinical examinations which were conducted at various facilities such as school halls, church halls, primary healthcare facilities, community centres and city halls. The blood samples were collected from individuals, aged 6 years and older, and used for biomarker analysis. The clinical examinations were conducted by experienced medical doctors and nurses on consenting individuals. The blood samples were collected and stored in cooler boxes and delivered to a laboratory within 24 hours. No deviations from established quality control measures were reported.

**Fig 1:**
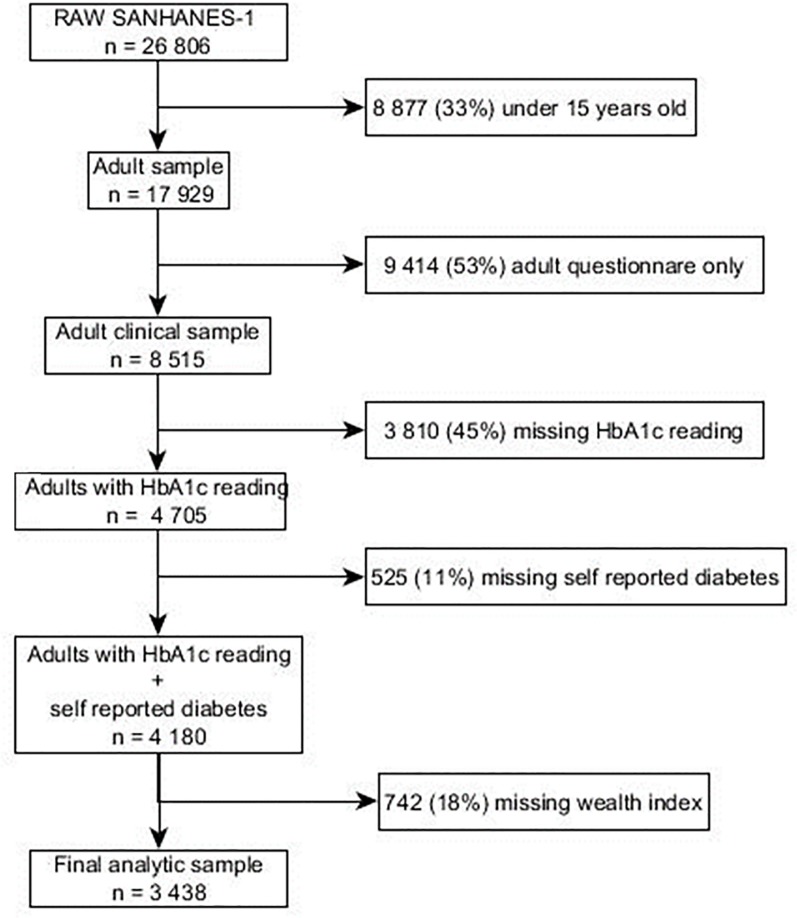
Flow chart for selecting the analytical sample from the general survey sample.

Our analysis is restricted to individuals above the age of 15 who had a blood sample taken and had non missing information on HbA1c; 17.8% had missing data on the wealth index and were also excluded. The final analytical sample used in this study is 3 438. Details of our exclusion criteria are shown in **Fig 1**.

### Measuring inequality

To measure inequalities in diabetes, this study makes use of the CI. The calculation of the CI requires a measure of socioeconomic status. In this study, a wealth index is used for this purpose. The wealth index was constructed with the aid of Multiple Correspondence Analysis (MCA). The household and living conditions considered in the creation of the wealth index are housing type, water and sanitation services, and a set of thirteen household assets. The full list of thirteen household assets is as follows: ownership of a fridge, television, stove, mobile phone, radio, DVD (digital video disc), washing machine, computer, DSTV (digital satellite television), motorcar, vacuum cleaner, telephone (landline) and internet access. Imputation by iterative binomial and multinomial logistic regression analysis, applied using Stata’s mi function, is employed to deal with item non-response. Asset ownership is imputed as a function of the ownership of the twelve other assets, whereas housing type is imputed from information on the material of the wall and roof of a dwelling. The percentage inertia explained by the first dimension is approximately 90%.

The CI is derived from the concentration curve (CC) which plots the cumulative percentage of the health variable against the cumulative percentage of the population ranked by the living standards measure [12] and the CI is measured as twice the area between the CC and the 45 degree line [12]. The CI takes on a value of zero when there is no socioeconomic related health inequality; which means that the health measure (in this case diabetes) is equally distributed across the population. It takes on a positive value when the health measure is more concentrated amongst the richer population and takes on a negative value when the health measure is more concentrated amongst the poorer population [12]. The magnitude of the CI indicates a disproportionate concentration of the health measure among the poor or the rich and takes on a value between +1 and -1. The CI can then be measured as follows: twice the covariance of the health variable and the ranking of the living standards variable r all divided by the mean of the health measure (*μ*):

$$CI=\frac{2}{\mu }cov(h,r)$$ (1)

Since all the health variables in our study are binary, a normalisation process is required to measure inequality. This study makes use of the Erreygers corrected concentration index which is algebraically expressed as shown below [25].

$$E(h)=\frac{4\mu }{b-a}CI$$ (2)

Where *μ* is the mean of the health variable, *CI* is the standard CI, b is the maximum value of the health variable (in this case 1) and *a* is the minimum value of the health variable (in this case 0). Similar to previous studies [26, 27] we made use of the *conindex* command in STATA to estimate inequalities.

### Decomposing socio-economic inequality

The CI can be decomposed into the factors that contribute to the measured inequality [28]. A review of the literature showed that there have been various developments in the methods applied in regression based decompositions of bivariate inequalities [28–31]. Whilst the Wagstaff decomposition technique [28] has been the dominant approach this method is one dimensional only [29, 31]. It ignores the correlation between health and the socioeconomic variable but rather focuses on the degree of variation in one variable only [29, 31]. Alternative methods have been suggested in the literature [29–31]. For example Erreygers and Kessels propose a two dimensional decomposition method that allows an analysis of the two variables (health and income) simultaneously [29]. To this Kessels and Erreygers introduced a structural equation modelling (SEM) approach which uses different sets of variables to explain the health and socioeconomic status variables. Heckley et al [31] makes use of the recentered influence function (RIF) regression approach developed by Firpo et al [32] to decompose the inequalities into their underlying determinants whilst addressing the limitations within the Wagstaff decomposition method. This approach however relies on a suitable identification strategy. Although such approaches do not ignore the bivariate nature of the bivariate rank dependent indices they have been commented on as being data demanding [31]. We adopt the dominant Wagstaff decomposition which also allows comparability with other studies that have used this method within the literature.

Following Wagstaff et al. [28] our health variable *h*_*i*_ (diabetes), is linked to a set of explanatory variable *x*_*ij*_ by the following linear model.

$$h_{i}=\alpha +\sum_{j=1}^{q}\beta_{j}x_{ij}+\varepsilon_{i}$$ (3)

If we have such linear model as shown in Eq (3) Wagstaff et al. shows that the concentration index for *h*_*i*_ can be written as [28]:

$$CI(h)=\sum_{j=1}^{q}\frac{\beta_{j}\bar{x_{j}}}{\mu_{h}}CI(x_{j})+\frac{{GC}_{\varepsilon }}{\mu_{h}}$$ (4)

In Eq (4), *CI*(*h*) is the CI for the health variable h (diabetes), $\bar{x_{j}}$ is the mean of *x*_*j*_, *μ*_*h*_ is the mean of the health variable, *CI*(*x*_*j*_) is the CI for *x*_*j*_, *GC*_*ε*_ is the generalised CI for the error term. In this equation the first part is the weighted sum of the CI for the variable *x*_*j*_. The weight of each regressor is determined by the elasticity ($\beta \bar{x_{j}}$) of h with respect to *x*_*j*_. The second part is the residual socio-economic inequalities in health that cannot be explained by the CI of the regressors. Since we applied the Erreygers normalisation to the calculation of the CI for the socio economic inequalities in diabetes, the corrected CI for the health variable is formulated as:

$$E(h)=4\sum_{j=1}^{q}\beta_{j}\bar{x_{j}}CI(x_{j})+{4GC}_{\varepsilon }$$ (5)

Eq (5) can now be used to decompose socio-economic inequalities in diabetes, showing the contribution of each factor. If the contribution of variable x is positive, then inequality in the health variable would decrease if variable x becomes equally distributed across the socio-economic group, ceteris paribus. The opposite is also true, if a contribution is negative, the absence of inequalities in that variable would result in an increase in inequality, ceteris paribus.

The absolute contribution a variable makes to socio-economic inequality is a product of the elasticity ($\beta \bar{x_{j}}$) of diabetes for each variable and the CI for each variable. Therefore, to estimate the contribution, we need to firstly estimate the coefficients of the explanatory variables via a regression. Ordinary Least Squares (OLS), Probit and Generalised Linear Models (GLM) are the three most common regression methods used for decomposition of inequalities [33]. Yiengprugsawan et al. compare these three decomposition approaches and show that the use of a GLM model (with binomial family and identity link) is the best choice when decomposing inequality of a binary variable [33]. Since our outcome variable is binary and following Yiengprugsawan et al. [33] and other studies [34, 35], this study makes use of the GLM model for decomposition of the Erreygers CI.

As there is no analytical expression for the computation of the standard errors for the contributions generated from Eq (4) and since the Stata bootstrap prefix command does not work [12, 36], a bootstrapping technique was used to generate the standard errors for the absolute contributions. Whilst taking into account the data’s sampling structure we applied the bootstrapping method as described in Efron et al. and Efron [37, 38] and applied in Ataguba et al. [36]. Bootstrapping allows us to assess sampling variability and obtain statistical inference on the results from the decomposition [39]. A total of 500 replications were used to estimate the standard errors.

Data analysis was conducted in STATA 13 and post-stratification sample weights were used in all analysis to adjust for unequal probabilities of selection and non-response.

### Diabetes indicators

From the analytical sample of 3 438, we identified five main diabetes health indicators: total, undiagnosed, diagnosed, treated and controlled diabetes.

#### 1. Total diabetes

**Fig 2:**
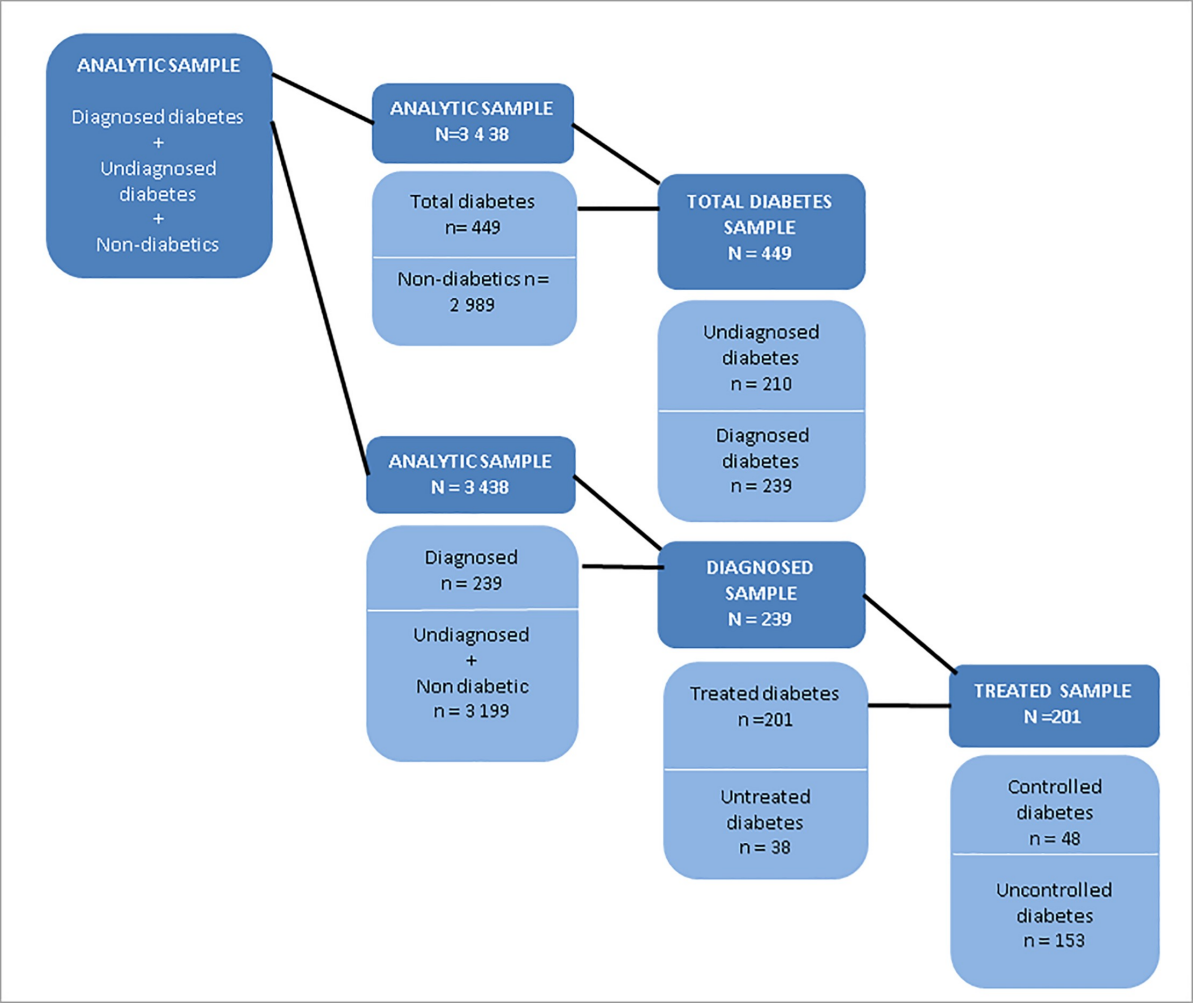
Health care categories sample sizes.

Total diabetes was defined as individuals who self-reported being diabetic or had undiagnosed diabetes. The self-reported and undiagnosed diabetes outcomes are explained in more detail below. A total diabetes binary variable was then created to estimate the socio-economic inequalities in total diabetes (see Fig 2). The binary variable took on the following values; 0 = individual did not have undiagnosed or self-reported diabetes, 1 = individual self-reported being diabetic or had undiagnosed diabetes.

#### 2. Undiagnosed diabetes

Among the sub-sample of total diabetics, we calculated the proportion of individuals with undiagnosed diabetes. According to the World Health Organisation the diagnostic criteria for diabetes is an HbA1c level greater than or equal to 6.5% [2]. Consistent with other studies, we defined diabetes as being undiagnosed when an individual did not self-report prior diabetes diagnosis by a physician, did not report currently taking any diabetic medication, and has a glucose test result of greater than or equal to 6.5% [40]. A binary variable is created to estimate inequalities in undiagnosed diabetes within the total diabetic sample (see Fig 2). The binary variable took on the following values; 0 = individual self-reported being diabetic, 1 = individual had undiagnosed diabetes.

#### 3. Diagnosed diabetes

Based on the SANHANES adult questionnaire, individuals were regarded as diabetic if they answered yes when asked if a medical doctor or other healthcare professional had told them that they have high blood sugar or if they answered yes when asked if they are currently taking insulin or tablets to lower their blood sugar levels. A binary variable was then created to estimate the socio economic inequalities in diagnosed diabetes (see Fig 2). The binary variable took on the following values; 0 = individual did not self-report being diabetic, 1 = individual self-reported being diabetic or taking diabetic medication.

#### 4. Treated diabetes

Among the diagnosed sample (self-reported diabetics), we calculated the proportion of diabetics that reported being on diabetic treatment. Diabetic individuals were considered to be taking treatment if they reported currently taking insulin or tablets to lower their blood glucose levels. A binary variable was created which took on the following values, 0 = if a self-reported diabetic individual reported not taking insulin or tablets for the lowering of blood glucose levels, and 1 = if a self-reported diabetic individual reported taking insulin or tablets for the lowering of blood glucose levels (see Fig 2).

#### 5. Controlled diabetes

Among the sample on diabetes treatment we calculated the proportion of individuals with controlled diabetes. Diabetes was defined as controlled if the respondent reported taking diabetes treatment (insulin or tablets) and had an HbA1c test of < 6.5%. A binary variable was then created for diabetes control amongst the treated sample, taking on the following values: 0 = individual was on diabetes treatment and had an HbA1c test of > 6.5%, 1 = if the individual was on diabetes treatment and had an HbA1c test of < 6.5% (see Fig 2).

### Explanatory variables–dietary, lifestyle and metabolic risk factors

A systematic review and meta-analysis by Aune et al. shows that all types of physical activity are beneficial in reducing the risks of type two diabetes [41]. Physical activity data was taken from the Global Physical Activity Questionnaire (GPAQ) within the SANHANES survey. To calculate the intensity of physical exercise, we multiplied weekly activity data of walking, moderate intensity activities and vigorous intensity activities by Metabolic Equivalents (MET) values of 3.3, 4.0 and 8.0 respectively [42]. The intensity of physical exercise variable (MET-minutes) was then used to create a categorical variable. The WHO recommendations on physical activity is achieving a minimum weekly exercise equivalent to 600 MET-minutes [43]. We categorised the physical activity variable as follows: 0–0 MET-minutes, 1 –> 0 < 600 MET-minutes, 2 - > = 600 < 2000 MET-minutes and 3 - > = 2000 MET-minutes. For unhealthy diet, two measures are used: consumption of fruits and of vegetables. Low fruit and vegetable consumption is referred to as the intake of fewer than five portions a day [44, 45]. Fruit and vegetable consumption is included as categorical variables that took on the values of 0 –none, 1 –less than four times a day, 2 –more than four 4 times a day. Evidence also suggests that smoking is associated with diabetes, however the increase in diabetes risk varies with smoking intensity [19]. In the SANHANES-1, respondents were also asked how many manufactured cigarettes they smoke per week, this was included as a continuous variable. Because alcohol is reported to have both beneficial and harmful effects, the association of alcohol and the risk of type 2 diabetes are influenced by alcohol drinking frequencies [20]. Alcohol consumption is therefore included as a categorical variable taking the values 0 –never, 1 –occasional and 2 –regularly. Body mass index (BMI) was calculated as weight divided by height squared and included as categorical variable that took on the values, 0 –underweight (BMI< 18.5), 1 –normal weight (BMI ≥ 18.5 and <25), 2 –overweight (BMI ≥ 25 and < 30) and 3 –obese (BMI ≥ 30).

### Other explanatory variables

Apart from the lifestyle factors and the wealth index, we also included a range of other variables which past literature has shown to influence health [46–48]. These variables include gender, residence, age, race, employment status, family history of diabetes, insurance and obesity. Gender was included as a binary variable 1 –male, 2 –female. Residence was included as a binary variable with 0 –urban and 1 –rural. Age was measured in years and included as a categorical variable, 15–35 years, 36–60 years and 61 + years. Race was included as a binary variable with 0 –African, 1 –Non-African (i.e. white, coloured and Indian). Employment was included as a binary variable, 0 –unemployed, 1 –employment. Family history of diabetes and insurance were both included as binary variables, 0 –No and 1 –Yes.

## Results

### Descriptive statistics

Table 1 shows survey weighted descriptive statistics for the study sample according to diabetes outcome. According to the data, the total prevalence rate of diabetes was 11% (total diabetes). Of the total diabetics, 38% were undiagnosed. The prevalence rate of self-reported diabetes was 7%. Of the self-reported diabetics, 61% were on treatment and 31% of those on treatment had controlled diabetes.

**Table 1:** Descriptive statistics of variables in study sample.

	Total Prevalence			Undiagnosed diabetes		Diagnosed diabetes		Treated		Controlled	
Variable	Mean	SE		Mean	SE	Mean	SE	Mean	SE	Mean	SE
	(n = 380; 11.09%)			(n = 170; 38.15%)		(n = 235; 6.86%)		(n = 145; 60.95%)		(n = 62; 31.28%)	
Sex											
Male	0.3296		0.0222	0.3048	0.0318	0.3450	0.0309	0.4409	0.0352	0.4716	0.0728
Female	0.6704		0.0222	0.6952	0.0318	0.6550	0.0309	0.5591	0.0352	0.5284	0.0728
Residence											
Urban	0.7775		0.0197	0.7383	0.0304	0.8017	0.0259	0.7491	0.0307	0.8088	0.0574
Rural	0.2225		0.0197	0.2617	0.0304	0.1983	0.0259	0.2509	0.0307	0.1912	0.0574
Age category											
15–35	0.1380		0.0163	0.1922	0.0273	0.1046	0.0199	0.0846	0.0197	0.1183	0.0471
36–60	0.5815		0.0233	0.5205	0.0346	0.6191	0.0315	0.5352	0.0354	0.3297	0.0686
61+	0.2806		0.0212	0.2874	0.0313	0.2764	0.0290	0.3802	0.0344	0.5520	0.0725
Race											
African	0.5344		0.0236	0.6302	0.0334	0.4753	0.0324	0.5620	0.0352	0.5161	0.0729
Non-African	0.4656		0.0236	0.3698	0.0334	0.5247	0.0324	0.4380	0.0352	0.4839	0.0729
Employment											
unemployed	0.5138		0.0239	0.5423	0.0351	0.4962	0.0328	0.5938	0.0351	0.6489	0.0704
employed	0.4862		0.0239	0.4577	0.0351	0.5038	0.0328	0.4062	0.0351	0.3511	0.0704
Diabetes history											
No	0.5463		0.0242	0.6147	0.0345	0.5063	0.0336	0.3525	0.0351	0.5455	0.0768
Yes	0.4537		0.0242	0.3853	0.0345	0.4937	0.0336	0.6475	0.0351	0.4545	0.0768
Insurance											
No	0.7280		0.0211	0.8931	0.0215	0.6260	0.0316	0.7526	0.0307	0.8216	0.0564
Yes	0.2720		0.0211	0.1069	0.0215	0.3740	0.0316	0.2474	0.0307	0.1784	0.0564
Obesity											
underweight	0.0142		0.0058	0.0067	0.0058	0.0190	0.0091	0.0188	0.0099	0.0162	0.0188
Normal weight	0.1439		0.0171	0.1215	0.0232	0.1582	0.0244	0.1701	0.0275	0.2148	0.0612
overweight	0.3802		0.0236	0.2408	0.0304	0.4692	0.0333	0.3259	0.0343	0.3785	0.0723
obese	0.4616		0.0242	0.6309	0.0343	0.3536	0.0319	0.4852	0.0365	0.3905	0.0727
Smoking Intensity (mean)	4.8973		0.9055	6.5562	1.5781	4.0890	1.1006	4.8758	1.3973	1.9057	2.7395
Alcohol cons											
No	0.8550		0.0168	0.8578	0.0244	0.8533	0.0232	0.8456	0.0258	0.9346	0.0364
Occasional	0.1118		0.0150	0.1095	0.0218	0.1133	0.0208	0.1072	0.0221	0.0413	0.0293
Regular	0.0332		0.0085	0.0327	0.0124	0.0335	0.0118	0.0472	0.0151	0.0241	0.0226
Physical activity											
None	0.3510		0.0226	0.3860	0.0337	0.3293	0.0305	0.4102	0.0349	0.3954	0.0713
Low	0.1047		0.0145	0.0830	0.0191	0.1180	0.0210	0.1423	0.0248	0.1720	0.0550
Moderate	0.1286		0.0158	0.1881	0.0270	0.0919	0.0188	0.1110	0.0223	0.1296	0.0490
High	0.4158		0.0233	0.3429	0.0328	0.4608	0.0324	0.3365	0.0335	0.3030	0.0670
Fruit consumption											
None	0.1092		0.0149	0.1486	0.0250	0.0851	0.0183	0.1177	0.0231	0.2010	0.0591
<4 times a day	0.8415		0.0175	0.8263	0.0266	0.8508	0.0234	0.8031	0.0285	0.7990	0.0591
4+ times a day	0.0493		0.0104	0.0251	0.0110	0.0641	0.0161	0.0793	0.0193	0.0000	0.0000
Vegetable consumption											
None	0.0335		0.0086	0.0288	0.0117	0.0363	0.0123	0.0594	0.0170	0.0925	0.0427
<4 times a day	0.7557		0.0206	0.9122	0.0198	0.6615	0.0311	0.8297	0.0270	0.9072	0.0428
4+ times a day	0.2108		0.0195	0.0591	0.0165	0.3022	0.0302	0.1109	0.0225	0.0003	0.0026

Note: All estimates are weighted proportions, SE-Standard error.

In each diabetes health outcome category the sample is predominantly female, resides in urban areas, is unemployed, has no health insurance and is overweight or obese. Our sample is predominantly within the age group of 36 to 60, with the exception of controlled diabetes. Approximately 63% of the undiagnosed sample was made up of Africans. The group that self-reported diabetes was predominantly non-African. However, the majority of respondents under treatment and with controlled diabetes were African. Based on the lifestyle factors, our sample predominantly consumed fruits or vegetable portions less than four times a day and did not consume alcohol. A majority of the individuals who self-reported diabetes did not drink alcohol, and conducted weekly exercise equivalent to more than 2 000 MET-minutes. With the exception of diagnosed diabetes a majority of the respondents within the health outcomes were obese.

### Diabetes prevalence across socio-economic groups

**Fig 3:**
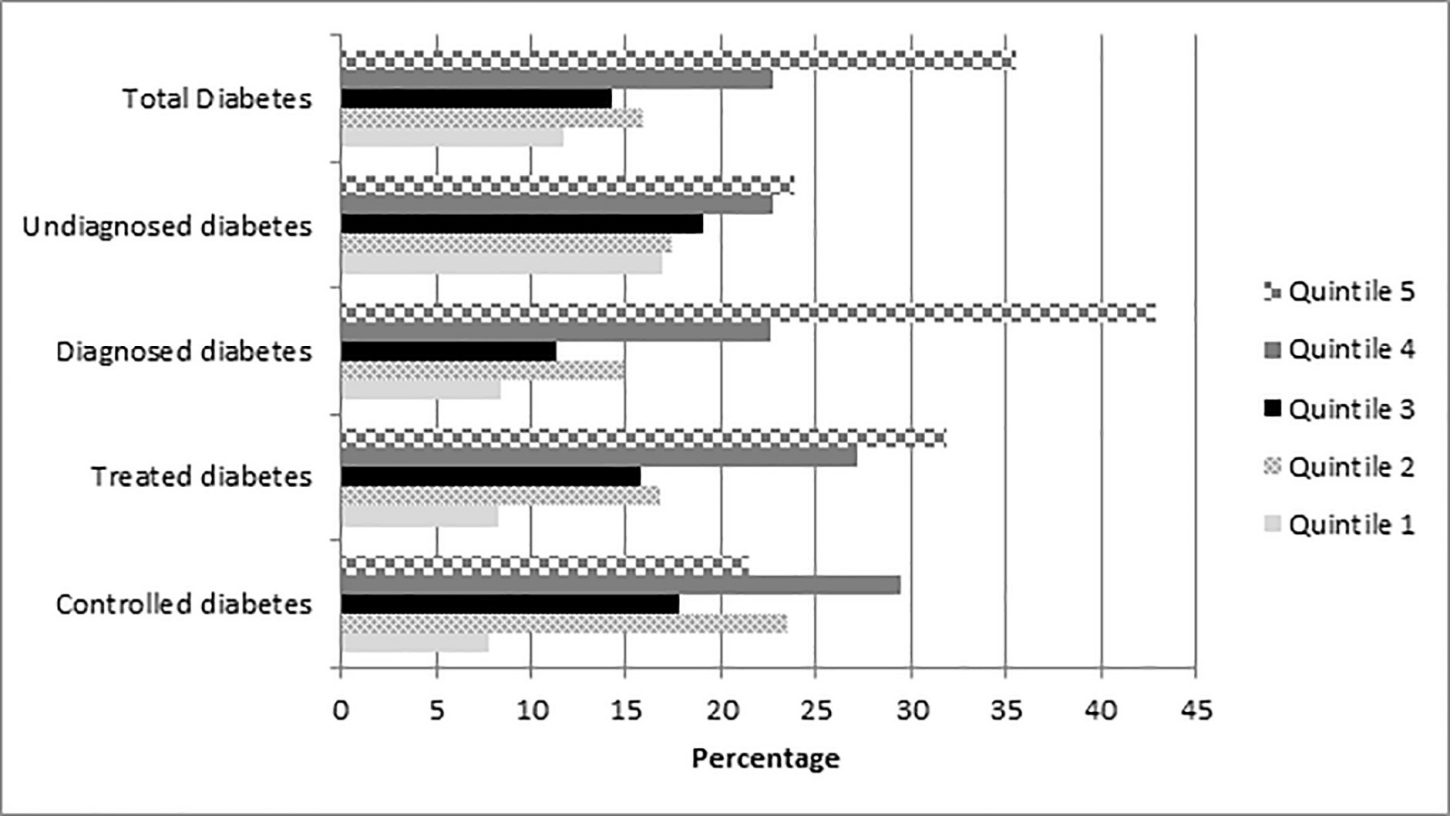
Distribution of diabetes by wealth quintile.

Fig 3 shows the distribution of diabetes categories by wealth quintile. From the graph it is clear that the distribution of all diabetes categories is not even across wealth index quintiles. Controlled diabetes was highest in the fourth quintile and all other outcomes were highest in the fifth quintile. All diabetes outcomes were lowest in the first quintile. The number of individuals with undiagnosed diabetes appeared to increase with wealth.

### Lifestyle factors prevalence across socio-economic groups

Table 2 shows that the distribution of lifestyle factors is not even across wealth index quintiles. The majority of respondents who drink alcohol are from the highest wealth quintile. The consumption of fruits and vegetables more than four times a day appears to increase with wealth. The majority of the individuals who do not consume any fruits or vegetables lie within the poorest quintile, whilst the majority that consume fruits and vegetables more than 4 times per week are within the richest quintile. Our results also show that across all wealth quintiles the majority of respondents conducted weekly exercise equivalent to more than 2 000 MET-minutes. Cigarette consumption intensity was highest within the highest wealth quintile.

**Table 2:** Distribution of lifestyle factor by wealth quintile.

Variable	Wealth Index Quantiles (%)				
	Poorest	Poor	Middle	Rich	Richest
Smoking (mean)	6.33	5.95	5.67	6.83	7.12
Alcohol consumption					
No	76.04	78.59	77.19	73.35	66.66
Occasional	15.82	17.87	17.95	20.84	19.2
Regular	8.14	3.54	4.86	5.81	14.13
Physical activity					
None	28.13	32.66	30.76	28.65	35.75
Low	12.19	15.97	12.83	14.27	8.98
Moderate	17.6	12.73	13.39	13.01	10.99
High	42.07	38.64	43.03	44.07	44.28
Fruit consumption					
None	20.36	17.05	9.8	9.96	6.64
<4 times a day	74.63	80.04	87.46	83.27	83.27
4+ times a day	5.01	2.91	2.74	6.77	10.09
Vegetable consumption					
None	9.85	7.54	3.68	6.52	2.29
<4 times a day	84.76	89.74	92.19	84.03	84.93
4+ times a day	5.39	2.72	4.13	9.46	12.78

Note: All estimates are weighted

### Concentration indices

Table 3 shows the corrected Erreygers CIs for each of the three diabetes outcomes. Due to the small sample sizes and loss of statistical power we do not present the CIs for controlled and treated diabetes. The results show that socio-economic inequality is statistically significant for self-reported and total diabetes. All statistically significant CIs are pro-rich, indicating a greater burden of self-reported and total diabetes amongst the higher socio-economic groups. The extent of inequality is worse in the total diabetes outcome when compared to the self-reported diabetes outcome. The concentration index for undiagnosed diabetes was calculated from a different sub-sample of the data and was pro-poor but not statistically significantly different from zero.

**Table 3:** Concentration indices for health categories.

Health variable	Erreygers CI	SE	P-value
Self-reported diabetes	0.0746	0.0348	0.0328
Undiagnosed diabetes	-0.1703	0.1257	0.1768
Total diabetes	0.0859	0.0352	0.0153

Abbreviation: SE—Standard error, CI—Concentration indices

### Decomposition of socio-economic inequality in diabetes

In order to better understand the lifestyle factors that contribute to inequalities in the diabetes outcomes we conducted a decomposition analysis. The decomposition analysis was conducted only for the measured inequalities in self-reported and total diabetes. Our study does not decompose inequalities in undiagnosed diabetes because our findings show that the measured inequalities in this diabetes outcome was statistically insignificant.

Table 4 displays the contribution of lifestyle factors to inequalities in self-reported diabetes and total diabetes. The table shows the margins, the elasticity (product of the coefficient and mean of each explanatory variable), the CI of the explanatory variables, the absolute, percentage and total contributions of lifestyle factors whilst also adjusting for other demographic and socio-economics variables. The table also presents the standard errors for the absolute contributions obtained via a bootstrapping method using 500 replications.

**Table 4:** Decomposition of inequality in self-reported diabetes and total diabetes.

	Self-reported diabetes								Total diabetes						
Variable	Margins		Elasticity	CI	Absolute	SE	%	Total	Margins	Elasticity	CI	Absolute	SE.	%	Total
Sex															
Male			(base)							(base)					
Female	-0.0307		-0.0163	-0.0205	0.0013	0.0021	1.79	1.79	-0.0392	-0.0208	-0.0205	0.0017	0.0027	1.98	1.98
Residence															
Urban			(base)							(base)					
Rural	0.0481**		0.0146	-0.4458	-0.0260	0.0212	-34.90	-34.90	0.0275	0.0083	-0.4458	-0.0149	0.0212	-17.33	-17.33
Age category															
15–35	0.0000		(base)						0.0000	(base)					
36–60	0.1103***		0.0411	0.0454	0.0075	0.0076	10.00		0.1357***	0.0505	0.0454	0.0092	0.0080	10.69	
61+	0.1474***		0.0174	0.0987	0.0069	0.0069	9.21	19.21	0.2107***	0.0249	0.0987	0.0098	0.0086	11.44	22.13
Race															
African			(base)							(base)					
Non-African	0.0158		0.0043	0.4808	0.0082	0.0183	11.05	11.05	0.0460*	0.0125	0.4808	0.0241	0.0206	27.99	27.99
Wealth index															
wealth1			(base)							(base)					
wealth2	0.0776**		0.0132	-0.4338	-0.0230	0.0280	-30.79		0.0770*	0.0131	-0.4338	-0.0228	0.0148	-26.53	
wealth3	0.0561		0.0120	-0.0492	-0.0024	0.0059	-3.17		0.0362	0.0077	-0.0492	-0.0015	0.0032	-1.78	
wealth4	0.0723**		0.0150	0.3717	0.0222	0.0251	29.81		0.0622	0.0129	0.3717	0.0192	0.0162	22.29	
wealth5	0.0784**		0.0165	0.7893	0.0522	0.0376	69.91	65.77	0.0428	0.0090	0.7893	0.0285	0.0253	33.11	27.10
Employment															
unemployed			(base)							(base)					
employed	0.0000		0.0000	0.1406	0.0000	0.0051	0.01	0.01	0.0118	0.0044	0.1406	0.0025	0.0054	2.86	2.86
Diabetes history															
No			(base)							(base)					
Yes	0.0719***		0.0178	0.1388	0.0099	0.0043	13.23	13.23	0.0668***	0.0165	0.1388	0.0092	0.0045	10.67	10.67
Insurance															
No			(base)							(base)					
Yes	0.0155		0.0028	0.6177	0.0068	0.0119	9.11	9.11	-0.0319	-0.0057	0.6177	-0.0140	0.0174	-16.27	-16.27
Obesity															
underweight			(base)							(base)					
normal weight	-0.0270		-0.0110	-0.1356	0.0060	0.0233	8.01		0.0019	0.0008	-0.1356	-0.0004	0.0276	-0.48	
overweight	0.0252		0.0063	0.1260	0.0032	0.0137	4.27		0.08702**	0.0219	0.1260	0.0110	0.0183	12.82	
obese	0.0639*		0.0173	0.1354	0.0094	0.0249	12.55	24.83	0.1357***	0.0367	0.1354	0.0199	0.0288	23.16	35.49
Smoking Intensity (mean)	-0.0015		-0.0097	0.0227	-0.0009	0.0050	-1.18	-1.18	-0.0027*	-0.0178	0.0227	-0.0016	0.0056	-1.87	-1.87
Alcohol cons															
No			(base)							(base)					
Occasional	-0.0274		-0.0050	0.0630	-0.0013	0.0014	-1.70		-0.0579***	-0.0106	0.0630	-0.0027	0.0024	-3.12	
Regular	-0.1123***		-0.0083	0.1766	-0.0059	0.0049	-7.89	-9.59	-0.1397***	-0.0104	0.1766	-0.0073	0.0050	-8.53	-11.65
Physical activity															
None			(base)							(base)					
Low	-0.0402		-0.0051	-0.0405	0.0008	0.0027	1.11		-0.0140	-0.0018	-0.0405	0.0003	0.0018	0.34	
Moderate	0.0163		0.0022	-0.0717	-0.0006	0.0022	-0.85		0.0059	0.0008	-0.0717	-0.0002	0.0022	-0.26	
High	0.0061		0.0026	0.0059	0.0001	0.0015	0.08	0.35	-0.0001	0.0000	0.0059	0.0000	0.0018	0.00	0.07
Fruit consumption															
None			(base)							(base)					
<4 times a day	0.0311		0.0255	0.0212	0.0022	0.0056	2.90		0.0260	0.0213	0.0212	0.0018	0.0091	2.10	
4+ times a day	-0.0056		-0.0003	0.1927	-0.0002	0.0099	-0.33	2.57	-0.0426	-0.0024	0.1927	-0.0018	0.0129	-2.14	-0.04
Vegetable consumption															
None			(base)							(base)					
<4 times a day	-0.0632*		-0.0551	-0.0027	0.0006	0.0026	0.78		-0.0199	-0.0174	-0.0027	0.0002	0.0026	0.21	
4+ times a day	0.0525		0.0037	0.2250	0.0033	0.0076	4.47	5.25	0.1684***	0.0119	0.2250	0.0107	0.0053	12.45	12.67
**Dietary, lifestyle and metabolic risk factors**								**22.24**	** **						**34.67**
**Total observed**		** **			**0.0803**							**0.0809**			
Residual					-0.0057							0.0050			
**Total**		** **			**0.0746**							**0.0859**			

As shown in Table 4, demographic and socio-economics variables that were significantly associated with self-reported diabetes were living in rural areas (p< = 0.05), increasing age (p< = 0.01), wealth (p< = 0.05) and family history of diabetes (p< = 0.01). Factors significantly associated with total diabetes were increasing age (p< = 0.01), being non-African (p< = 0.1), wealth quintile 2 (p< = 0.1) and a family history of diabetes (p< = 0.01). Factors that contributed the most to socioeconomic inequalities in both self-reported and total diabetes were residence (urban or rural dwelling), wealth index (socio-economic status) and age. Our results show that residence explains -34.9% of the inequality in self-reported diabetes and -17.3% of the inequality in total diabetes. Thus, if inequalities in diabetes were determined by this variable alone they would favour the better off. According to Table 4 the wealth index is also a significant contributor to self-reported diabetes (65.77%) and total diabetes (27.10%).The contribution of the wealth index to diabetes inequalities is higher for self-reported diabetes compared to total diabetes because of the different elasticity values. Age category was also another large contributor to inequality, contributing 19.2% to inequalities in self-reported diabetes and 22.1% to inequalities in total diabetes. Race and family history of diabetes also make notable contributions to inequality.

The marginal effects in Table 4 show that the lifestyle factors significantly associated with self-reported diabetes were being obese (p< = 0.1), regular alcohol consumption (p< = 0.01) and vegetable consumption less than four times a day (p< = 0.1). Factors significantly associated with total diabetes were obesity (p< = 0.05), smoking (p< = 0.1), alcohol consumption (p< = 0.01) and vegetable consumption more than four times a day (p< = 0.01). Results from the decomposition show that lifestyle factors contributed a total of 22.2% and 34.7% to inequalities in self-reported and total diabetes respectively. Among the lifestyle factors, obesity, alcohol and vegetable consumption made the largest contribution to diabetes inequalities (see Table 4). Obesity contributed approximately 24.8% to inequalities in self-reported diabetes and 35.5% to inequalities in total diabetes. In the absence of inequalities in obesity, inequalities in diabetes would decrease. Vegetable consumption is another important contributor to diabetes inequalities, 5.3% for self-reported diabetes and 12.7% for total diabetes. The positive contribution by vegetable consumption indicates that if vegetable consumption was equally distributed across the wealth index then inequalities in self-reported diabetes would decrease by 5.3% and 12.7% for total diabetes. Alcohol consumption contributed -9.6% to inequalities in self-reported diabetes and -11.7% to inequalities in total diabetes, meaning that if alcohol consumption was distributed equally amongst the population, inequalities in self-reported and total diabetes would increase. Smoking intensity, fruit consumption and physical activity made marginal contributions to diabetes inequalities. The residuals in Table 4 represent the unexplained sources of inequalities.

## Discussion

Our paper provides evidence on the socio-economic inequalities in various diabetes outcomes using the CI and identifies the contribution of lifestyle factors to socio-economic inequalities in diabetes prevalence by conducting a decomposition analysis. To the best of our knowledge this is the first paper to incorporate biomarker analysis in the measurement of diabetes inequalities in South Africa and the first to attempt to measure the contribution of various lifestyle factors to socio-economic related inequalities in diabetes. Consistent with the study by Stokes et al., our study documents the high levels of undiagnosed diabetes in South Africa [18]. This study showed that the total prevalence of diabetes in South Africa was 11%, of which 38% were undiagnosed. The poor rates of diagnosis are largely a result of insufficient access to health care and poor health systems [17]. The prevalence of self-reported diabetes was 6.86% of which 61% were on treatment and 31% of those on treatment had controlled diabetes. The poor rates of treatment and control have also been previously reported by Stokes et al. [18] and have been attributed to poor diabetes education and medication adherence [17, 18].

Our findings corroborate other related literature that demonstrates the existence of socio-economic inequalities in diabetes [13, 15]. Furthermore, consistent with previous studies that estimated inequalities in self-reported diabetes in South Africa our study finds that the prevalence of self-reported diabetes is pro-rich [13, 15]. Although our findings on the inequality in undiagnosed diabetes were not statistically significantly different from zero, we find that the size of the inequality in diagnosed diabetes was further intensified by the inclusion of undiagnosed diabetics. This finding informs the development of studies that seek to produce more robust inequality estimates of NCD prevalence in South Africa. The finding also contributes to international literature that has attempted to use the concentration index to compare the use of self-reported diagnosis versus standardised measures of diagnosis in the estimation of socioeconomic inequalities in health [16, 49]. Although the NCDs considered in these study excluded diabetes, the direction of inequality between self-reported chronic diseases and standardised measures of chronic disease diagnosis showed a mixed picture that varied by disease type and country [16, 49].

The decomposition of inequalities has become an important tool in influencing policy in inequality studies. Decompositions provide important information on the sources of the observed inequalities. Whilst the largest contributions to the inequalities in diabetes in this study came from residence and socio-economics status, the contributions of lifestyle factors further exacerbate these inequalities and are the focus of this analysis. Various studies have attempted to estimate the contribution of lifestyle factors to health in general [15, 50–52] and diabetes specifically [21, 22, 53]. A study by Borg and Kristensen showed that lifestyle factors and work environment contribute approximately two thirds to the social gradient in self-reported health [50]. Our study shows that lifestyle factors contributed a total of 22.2% and 34.7% to inequalities in self-reported and total diabetes, respectively. Previous studies suggest that these factors explain between 33–45% of inequalities in the incidence of type 2 diabetes in the United Kingdom [21], a third of socioeconomic inequalities in type 2 diabetes in a Swedish based study [22] and 27% when estimated using the Australian Diabetes Obesity and Lifestyle Study [53].

Amongst the lifestyle factors in our study obesity makes the largest contribution to socio-economic inequalities in both self-reported and total diabetes variable. Stringhini et al, using data from the London Whitehall II Study also finds that amongst the health behaviours in the study obesity was the most important contributor to the relationship between socio economic status and diabetes [21]. Obesity is widely regarded as a risk factor for ill health [23] and type 2 diabetes [2, 54]. Consistent with a study by Alaba and Chola, using the South African National Income Dynamics Study (NIDS), we observe a pro-rich distribution of disparities in obesity [55]. Our findings show a much larger contribution of obesity to self-reported and total diabetes (24.8% and 35.5%) than the contribution of obesity to social inequalities in health reported in the London Whitehall II study (18% - 20%%) [51]. South Africa is reported to be undergoing an epidemic of overweight and obesity that is closely linked to nutrition changes [56], severely impacting health outcomes.

Although evidence suggests that diets rich in fruits and vegetable are associated with a reduced risk of type 2 diabetes [57–59], the mechanisms through which fruits and vegetables consumption influences the diabetes risk is not well established [59]. Whilst some studies showed that the consumption of fresh fruit [58] was associated with a reduction in the risk of type 2 diabetes other literature shows that the reduction in risk is related to the fruit or vegetables sub-types consumed [60]. In particular dietary fibre is reported to regulate insulin which helps reduce diabetes risk [61] and green leafy vegetables are inversely related to diabetes [59, 62]. The observed differences are likely a result of the use of food frequency questionnaires rather than biomarkers (such as vitamin C) [59]. In our study vegetable consumption was associated with diabetes prevalence and contributed more to socio-economic inequalities in diabetes when compared to fruit consumption. Consistent with previous literature we find that lower consumption of fruits and vegetables is concentrated amongst those within low socio-economic groups [63, 64]. In South Africa factors such as urban migration and globalisation are reported to be the cause of the nutrition transition that has resulted in the consumption of energy-dense foods and sugary beverages [56].

Findings within the literature on the association between alcohol and diabetes have not been consistent [20]. Some studies report a protective effect at moderate consumption levels [20, 65], an increased risk at high consumption [65], a protective effect even at high consumption [66] and other studies find that the risk of diabetes in high alcohol consumers is the same as in abstainers [65]. Our study finds that regular and occasional alcohol consumption was negatively correlated with diabetes. Overall the rates of regular or occasional alcohol consumption in our study are quite low (25%), diabetics in our sample were less likely to drink alcohol. Alcohol consumption is reported to have both beneficial and harmful effects on health. Thus, the impact of alcohol consumption on health is a function of the length, volume, patterns and type of alcohol consumed. In our study alcohol made one of the largest contributions to inequalities in diabetes amongst the lifestyle factors. Contrary to other studies we find much larger contributions of alcohol to inequalities in diabetes [15, 21].

In our study smoking and physical activity make the smallest contributions to inequalities in diabetes. This is a result of the very small marginal effects and elasticities. Similar to the existing literature [15], we also found no evidence that smoking contributes significantly to inequalities in diabetes, while contrary to the literature, we did not find any evidence that physical activity explains a substantive proportion of inequalities in diabetes [22]. These differences most probably are attributable to differences in behavioural and situational contexts in different countries and settings.

## Study strengths and limitations

A major strength of this study is that it made use of an HbA1c test, an objective measure of diabetes. This measure allowed us to measure the prevalence of undiagnosed and total diabetes. The study has some limitations that must be acknowledged. Whilst there are several regression based decomposition methods within the literature our study makes use of the Wagstaff method. Results may differ depending on the decomposition method applied [31]. The American Diabetes Association states that although the risk of developing diabetes increases with age, there is no exact age for the onset of type 1 or type 2 diabetes, thus we were unable to separate type 2 from type 1 diabetics [67]. Despite this, lifestyle factors such as alcohol, physical activity and fruit consumption which are common risk factors for type 2 diabetes were included in our analysis as explanatory variables. Another limitation of the study is the low number of individuals who went to the testing centres and provided a blood sample. Our analytical sample may be prone to self-selection of individuals that went to get blood samples taken as well as those who completed the adult and household questionnaires. We therefore compared our final analytical sample to the 2011 South African census across sex, age, race and province. Compared to the 2011 census our analytic sample contained a larger sample of non-Africans (27% versus 23) and a smaller sample of Africans (72% versus 77%). Our sample also contained fewer individuals within the age category of 15 to 35 years (51% versus 55%). The analytical sample employed in this study therefore is not nationally representative, which means caution is necessary in drawing generalisations from the empirical results. It is also possible that our self-reported data on lifestyle factors suffered from social desirability bias. For example an under reporting of smoking patterns or alcohol consumption could potentially influence the contributions made by these factors to diabetes inequalities.

## Conclusion

This paper provides an analysis of the socio-economic inequalities in the prevalence of diabetes and determines the sources of these inequalities with a focus on modifiable lifestyle factors. The paper contributes to the literature on diabetes by making use of a more objective measure of diabetes and highlighting the magnitude of undiagnosed diabetes in South Africa. The study provides evidence that inequality in self-reported and total diabetes is concentrated among the rich. The magnitude of inequality estimates based on self-reported data only would be different when compared to inequality estimates based both on self-reported plus clinical data. The measured inequalities are mostly explained by residence and wealth. The contributions made by lifestyle factors to inequalities in diabetes, are less than the overall contributions of other factors within our model. Although modest, the contributions made by lifestyle factors to inequalities in diabetes provide important information for use in planning of interventions to reduce the burden of diabetes. Our study shows that in comparison to all other lifestyle factors obesity, alcohol consumption and vegetable consumption make large contributions to inequalities in diabetes. These findings are important to policy makers in terms of informing the design of effective strategies and policies for encouraging healthy lifestyles. Future national health surveillance surveys that capture larger numbers of individuals who provide blood samples are an ideal conduit for the monitoring of diabetes and the tracking of socio-economics inequalities in the prevalence, diagnosis and treatment of diabetes.

## Abbreviations

BMI: Body Mass Index
CI: Concentration Index
CC: Concentration Curve
DSTV: digital satellite television
EA: Enumeration area
GLM: Generalised Linear Model
MCA: Multiple Correspondence Analysis
MET: Metabolic Equivalents
NCDs: Non-Communicable Diseases
WHO: World Health Organisation
HSRC: Human Sciences Research Council
SANHANES-1: South African National Health and Nutrition Examination Survey
SES: Socio-economic Status
SDG: Sustainable Development Goals
VP: Visiting Points
GLM: Generalised Linear Model
